# Silicone Oil and Iodine-125 Brachytherapy for Uveal Melanoma in High-Risk Patients

**DOI:** 10.7759/cureus.5270

**Published:** 2019-07-29

**Authors:** Lance J Lyons, Ethan D Hinds, Sarada Chexal, Brian Berger

**Affiliations:** 1 Ophthalmology, University of Texas Medical Branch, Galveston, USA; 2 Ophthalmology, Retina Consultants of Austin, Austin, USA

**Keywords:** choroidal melanoma, plaque brachytherapy, radiation retinopathy, silicone oil

## Abstract

Purpose

Silicone oil a burgeoning adjuvant in the treatment of uveal melanoma where it is used for tissue protection during I-125 brachytherapy. While risk factors in the development of radiation retinopathy (RR) have been identified, treatment modulation for high-risk patients has largely been overlooked. We seek to expand the literature on this subject by reporting outcomes of I-125 brachytherapy with silicone oil in a high-risk population in the community setting.

Methods

Five patients with uveal melanoma and at least one risk factor for RR development underwent iodine-125 (I-125) plaque brachytherapy with concurrent pars plana vitrectomy (PPV), silicone oil administration, and fine needle aspiration biopsy (FNAB). Plaque and silicone oil removal were performed after seven days. Minimum follow-up was 12 months.

Results

Follow-up ranged from 12 to 56 months. Macular radiation doses ranged from 12.55 to 141.5 Gy; the two eyes with the largest doses developed RR at 34 and 15 months as well as neovascular glaucoma (NVG). Surgical complications included one rhegmatogenous retinal detachment (RD) and an intra-operative vitreous hemorrhage with post-operative hyphema requiring additional intervention.

Conclusion

RR may be attenuated by silicone oil administration in patients with some risk factors. In tumors farther from the macula, this benefit is more readily apparent. Tumors located more posteriorly may not benefit from silicone oil administration considering postoperative complications and operating time. Patient demographics, tumor characteristics, and anticipated macular radiation dosage may help determine which patients can benefit from silicone oil and identify patient risks for adverse outcomes.

## Introduction

The most common cause of primary intraocular cancer in adults is melanoma arising from the choroid and ciliary body [1]. The standard of care for small and medium-sized tumors is iodine-125 plaque (I-125) brachyotherapy, as established by the collaborative ocular melanoma study (COMS) [2-4]. However, patients generally lose two lines of visual acuity per year and nearly 45% develop acuity ≤20/200 within three years [5]. Side effects typically occur one and a half to two years post-treatment, though they may occur up to five years post-treatment [6,7]. Cystoid macular edema, macula ischemia, and chorioretinal atrophy constitute radiation retinopathy (RR), appearing in up to 43% of patients within five years of receiving brachytherapy [6,8]. In the COMS literature, photographic and angiographic studies confirmed radiation-associated macular pathology in up to 75% of patients in five years post-brachyotherapy. The high rate of adverse events indicates a need for improvement of radioactive brachytherapy for uveal melanoma.

Since 2010, various fluids have been considered to reduce radiation exposure to healthy tissues [9]. Of the four intraocular vitreous substitutes tested, 1000 and 5000-centistoke (cSt) silicone oil (polydimethyl-n-siloxane) were identified as candidates for further study due to the reduction of radiation penetrance by 48% and 47% under vitro and ex vivo conditions, respectively. A monte carlo simulation projected up to 57% reduction of radiation exposure to vital ocular structures. Ahuja et el. confirmed reductions of 65% in dose delivery under in vitro conditions, which prompted them to test the procedure in three patients [10]. Absence of RR up to to 24 months suggested that silicone oil may improve outcomes in clinical settings and opened the door to more robust studies.

In two case-controlled studies of 20 patients, McCannel et al. elucidated silicone oil’s potential role in treatment of uveal melanoma [11,12]. The 2014 study concluded that the experimental group yielded fewer abnormal maculas, lower central macular thickness on optical coherence tomography (OCT), and better final visual acuity. Two years later, they found that radiation attenuation from silicone oil significantly improved visual acuity.

Despite the recent success of silicone oil administration, high-risk patients have been overlooked in the literature. Identified risk factors for RR include tumors size, total radiation dose, proximity of tumor to affected structures, and systemic conditions such as diabetes mellitus [8,12-14]. Given the potential benefits of silicone oil administration, we sought to study outcomes in a patient sample otherwise considered as high-risk in a community setting, which may more closely reflect the population.

## Materials and methods

Inclusion criteria for patients were diagnosis of uveal melanoma with at least one known risk factor, including age over 60, systemic disease (hypertension and/or diabetes), or high macular radiation dose. Minimum follow-up was 12 months.

Five patients with medium to large sized melanomas (as defined by COMS guidelines) received I-125 plaque brachytherapy implanted by the same surgeon (BB) from 2011 to 2016 [15]. Dosimetry was 85 gray (Gy) to tumor apices, as determined by the Eye Physics^TM^ plaque simulator treatment plan. A 23-gauge trocar system established access to the posterior segment of the eye 4 mm from the limbus. Tumor location was confirmed using indirect ophthalmoscopy and transillumination. Fine needle aspiration biopsy (FNAB) was performed via a transcleral or transvitreal approach for cytopathology and molecular analysis. Core and peripheral vitrectomy were performed, followed by administration of either 1000 or 500 cSt silicone oil into the posterior segment. When necessary, extraocular muscles were displaced to achieve tumor coverage. An Eye Physics^TM^ plaque was then secured to the sclera. After seven days, the plaque and silicone oil were removed in a single procedure. When possible, barrier laser photocoagulation was applied to the tumor circumference during plaque removal. FNAB samples were sent for classification and risk stratification analysis. RR was determined clinically during follow-up.

## Results

Clinical charts were reviewed for patient demographics, tumor location and grade, and surgical details of brachytherapy (Table 1). Outcomes including eye retention, visual acuity, RR presence and time to development, toxicity, metastasis, and surgical complications were recorded (Table 2).

**Table 1:** Relevant Patient Demographics, Tumor Position, Macular Doses and Follow Up Time *Abbreviations: PMH- past medical history. H- height. L- length. W- width. HTN- hypertension. DM: type II diabetes mellitus. Gy- gray. mm- millimeters.

Patient	Age	Gender	PMH	Tumor Size (mm)	Tumor Location	Macular Dose	Distance from plaque to macula	Total Follow Up
1	60	Male	DM, HTN	H 11.02 L 9.7 W 9.41	Encroaching on macula inferotemporally	141.5 Gy	8.8 mm	56 months
2	61	Male	DM, HTN	H 6.2 L 12.0 W 12.0	Inferotemporal	21.25 Gy	16.77 mm	45 months
3	84	Female	HTN	H 3.18 L 12.92 W 8.87	Nasal	12.55 Gy	17.17 mm	26 months
4	68	Male	DM	H 7.3 L 13.6 W 14.5	Temporal	43.17 Gy	14.08 mm	12 months
5	39	Female	None	H 5.6 L 11.44 W 10.62	At superior macula	118.5 Gy	6.9 mm	51 months

**Table 2:** Outcome Measures *Abbreviations: HM- hand motion. NA- not applicable. CF- counts fingers. LP- light perception. NVG- neovascular glaucoma. RD- retinal detachment.

Patient	Visual acuity pre-op, post-op 1 year, 2 years, 3 years	RR, Time to Development	Other Toxicity or Complication	Surgical Complications
1	20/60 +2, HM, LP, HM	Yes, 34 months, with subsequent NVG	Optic atrophy, choroidal atrophy	Vitreous hemorrhage intra-op. Post-op hyphema
2	20/20, 20/20 -1, 20/40-2, 20/30	No	None	Rhegmatogenous RD (treated with full recovery of vision)
3	20/40 +1, 20/30 -2, 20/30 -1, NA	No	None	
4	20/50 -1, 20/30 -2, NA, NA	No	None	
5	20/125, 20/60 -1, CF@6ft, LP	Yes, 15 months, with subsequent NVG	Exudative RD, eventually enucleated	

Patient age at the time of diagnosis ranged from 39 to 84 years (median 61 years), and all patients had at least one risk factor for RR development. Follow-up ranged from 12 to 56 months (median 45 months). FNAB was performed without complication on all specimens and sent for tumor classification. Grade 1A was assigned to four out of five specimens, while Patient 3’s sample was grade 2. Two patients had tumors encroaching on the macula, while one was nasal to the posterior pole and two were greater than 13 mm temporal to the fovea. Tumor thickness ranged from 3.18 to 11.02 mm (median 6.2 mm).

Macular radiation dose ranged from 12.55 Gy to 141.5 Gy (median 43.17 Gy). Patients 1 and 5 developed RR at 34 and 15 months, respectively. Their tumors were located closest to the posterior pole. Thus, they received the two highest macular doses in the group (141.5 Gy and 118.5 Gy, respectively). Patient 1 also developed optic and choroidal atrophy secondary to radiation, while patient 5 developed extensive exudative RD. For eye salvage, Patient 5 received PPV with 360-degree retinectomy, endodrainage, endolaser, and 1000 cSt silicone oil injection at another institution.

Patients 1 and 5 developed RR and NVG. Patient 1 was treated with latanoprost and brimonidine/timolol but ultimately required tube-shunt surgery, which reduced the intraocular pressure to normal values. He retained visual acuity to hand motion. Patient 5 received intravitreal bevacizumab for iris neovascularization but was later enucleated due to pain. No patient developed melanoma recurrence, and there were no deaths attributed to tumor metastases.

Patients 1 and 2 developed surgical complications related to PPV. Patient 1 had vitreous hemorrhage during initial vitrectomy; after removal of silicone oil he had recurrent hyphema which was successfully treated with anterior chamber washout and intracameral bevacizumab. Patient 2 developed a rhegmatogenous RD three months after PPV and plaque placement. PPV, sulfur hexafluoride gas, and endolaser produced full recovery of vision.

## Discussion

Our case series fills a critical gap in the available literature by describing outcomes of patients with at least one risk factor for RR, after treatment for uveal melanoma with silicone oil and I-125 plaque administration. Male gender, age greater than 60 years, and systemic diseases such as hypertension and diabetes have been linked to higher risk of developing RR [16-18]. Other risk factors include tumor thickness greater than 4 mm and radiation doses above 90 Gy to the macula [19]. Despite these known risk factors, the three prior clinical studies with this method have not reported patient demographics in a standardized manner. Only one study has reported systemic disease, with three out of 40 study participants having diabetes [12]. In our study, four of five patients had hypertension or diabetes (two with both conditions). Four were older than 60 years at time of diagnosis while three patients were male. Four patients also had tumors greater than 4 mm thick and two had radiation doses above 90 Gy to the macula. We hope that future studies in this burgeoning field will consider risk factors due to patient demographics, tumor characteristics, and dosimetry.

Despite the risks, only two eyes developed RR over the course of the study. Those patients had tumors located in the posterior pole and thus received the highest macular doses of radiation. Tumors located posteriorly may not receive as much macular protection as those located more peripherally due to the oil’s inability to occlude radiation. Patients with macula-encroaching tumors and other risk factors for developing RR might be better served without vitrectomy and silicone oil insertion given their overwhelming risk for developing RR, the inability of silicone oil to block radiation to areas directly under the plaque, and the surgical complications that come with the additional procedure.

As silicone oil administration as an adjuvant to I-125 brachytherapy becomes increasingly adopted, we hope clinicians can develop an algorithm for risk stratification and prognosis. We agree with others that a preventative approach to radiation dose reduction may be an ideal approach, given that laser and intravitreal pharmacologic agents have failed to demonstrate efficacy [11,20]. In time, application of protective fluids such as silicone oil may become the standard during treatment of uveal melanoma.

## Conclusions

RR may be attenuated by administration of silicone oil in patients with some risk factors; in tumors farther from the macula, this benefit is more readily apparent. Tumors located more posteriorly may not benefit, considering postoperative complications and operating time. Patient demographics, tumor characteristics, and anticipated macular radiation dosage may help determine who can benefit from silicone oil and patient risks for adverse outcomes.
